# Investigation of LRS dependence on the retention of HRS in CBRAM

**DOI:** 10.1186/s11671-015-0771-0

**Published:** 2015-02-11

**Authors:** Xiaoxin Xu, Hangbing Lv, Hongtao Liu, Qing Luo, Tiancheng Gong, Ming Wang, Guoming Wang, Meiyun Zhang, Yang Li, Qi Liu, Shibing Long, Ming Liu

**Affiliations:** Laboratory of Nano-Fabrication and Novel Devices Integrated Technology, Institute of Microelectronics, Chinese Academy of Sciences, #3 Beitucheng West Road, Chaoyang District Beijing, 100029 China

**Keywords:** Resistive random access memory (RRAM), High resistance state (HRS), Retention, Quantum point contact (QPC) model

## Abstract

The insufficient retention prevents the resistive random access memory from intended application, such as code storage, FPGA, encryption, and others. The retention characteristics of high resistance state (HRS) switching from different low resistance state (LRS) were investigated in a 1-kb array with one transistor and one resistor configuration. The HRS degradation was found strongly dependent on the LRS: the lower the resistance of the LRS (*R*_LRS_) is, the worse HRS retention will be. According to the quantum point contact model, the HRS corresponds to a tiny tunnel gap or neck bridge with atomic size in the filament. The degradation of HRS is due to the filling or widening of the neck point by the diffusion of copper species from the residual filament. As the residual filament is stronger in case of the lower *R*_LRS_, the active area around the neck point for copper species diffusion is larger, resulting in higher diffusion probability and faster degradation of HRS during the temperature-accelerated retention measurement.

## Introduction

Resistive random access memory (RRAM), relying on the reversely switching between high resistance state (HRS) and low resistance state (LRS), has been widely explored as one of the most promising candidates due to its key advantages, such as simple structure, scalability potential, and complementary metal-oxide-semiconductor (CMOS) compatibility [1-6]. Nevertheless, the deficient switching mechanisms and ungenerous reliability still present a significant barrier to implement RRAM into large-scale commercial applications. Cation-based electrochemical metallization cell (ECM) and anion-based valence change memory (VCM) are two main categories of RRAM [1]. The ECM device also called as conductive bridge random access memory (CBRAM), whose switching mechanism is governed by creation (SET operation) and dissolution (RESET operation) of the metallic conductive filament (CF), likely related with the active metal species such as Ag or Cu [5,6]. On the other hand, the switching behavior of VCM is triggered by the drift of oxygen anions [7,8]. The copper-based CBRAM, which takes the advantage of the copper plug or copper line as the electrode, is a favorable choice with respect to cost-effective fabrication [9-11]. According to the CF model of CBRAM, the data retention is related with the diffusion of metal species [12,13]. In the case of LRS, the lateral diffusion of metal ions out of the CF causes a decrease of CF diameter or metal concentration in the CF, resulting in the degradation of LRS retention, whereas the degradation of HRS retention stems from the reconstruction of CF due to the vertical ions diffusion from the residual CF to the rupture region [12-14]. It has been reported that the retention of HRS or LRS was highly dependent on the resistance value of HRS or LRS, respectively [14-16]. The activation energy required for metal species diffusing in the electrolyte also affected the HRS and LRS retention greatly [16-22]. However, none of the prior arts has addressed the topic of LRS-dependent HRS retention yet.

In this work, we investigated the influence of LRS on HRS retention of CBRAM in 1-kb one transistor and one resistor (1T1R) array. The HRS cells, programmed from different LRS, were baked on vacuum oven under elevated temperature for retention measurement. We found the HRS retention was strongly dependent on the LRS: The lower *R*_LRS_ was, the worse HRS degradation would be. This behavior was explained by a comprehensive physical model based on metal diffusion mechanism.

## Method

**Figure 1:**
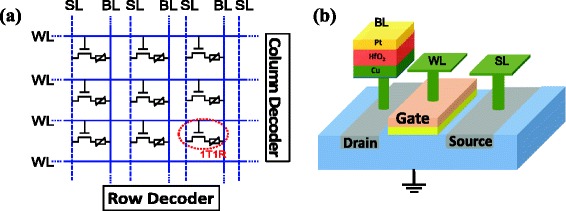
**The schematic of the 1T1R architecture and 1-kb array. (a)** The 1-kb 1T1R array was fabricated by standard 0.13-μm CMOS technology. **(b)** The 1T1R architecture was formed by integrating the RRAM stack on the copper plug.

A 1-kb array of N-MOS transistor was fabricated by standard 0.13-μm CMOS technology, followed by integrating Cu/HfO_2_/Pt RRAM stack on the copper plug on the drain terminal of the transistors, as shown in Figure 1. The HfO_2_ and Pt were about 4 and 70 nm, respectively, deposited by ion beam spurting. The size of RRAM cell was about 300 nm × 400 nm, defined by the area of copper plug. Finally, the 1T1R array was covered by a SiO_2_ passivation layer, leaving the test pads exposed. Electrical measurements were performed using Keithley 4200 analyzer. HRS retention characteristics were measured by baking the samples in a vacuum oven at an elevated temperature of 150°C, whose resistances were then checked periodically by a self-made array test system after device cooling down to room temperature.

## Results and discussion

**Figure 2:**
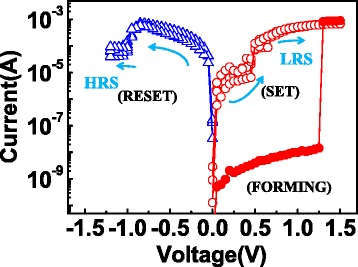
**The typical I-V characteristics of the 1T1R structure during FORMING and SET/RESET operations.** The memory cells show the bipolar resistive switching.

**Figure 3:**
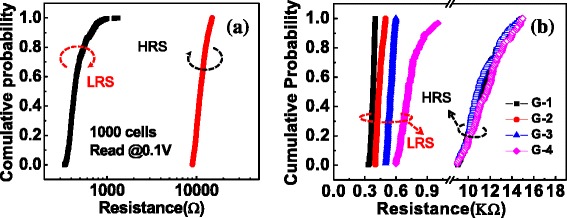
**The global statistical distribution of** ***R***_**LRS**_ **and** ***R***_**HRS**_ **. (a)** *R*_LRS_ is distributed in a range of 300 to 1,000 Ω, whereas *R*_HRS_ concentrates around 10 kΩ approximately. **(b)** *R*_LRS_ was divided into four different groups. The open-symbol lines: *R*_HRS_ distribution for four-group *R*_LRS_.

During the FORMING/SET operation, the wordline (WL) was forced by a positive bias to open the access transistor and provide compliance current. Positive sweeping voltage was applied on the sourceline (SL) to switch the memory cells from HRS to LRS with bitline (BL) kept on ground and vice versa for the RESET operation. Typical current–voltage (*I-V*) switching characteristics (FORMING, SET, and RESET) in DC modes are illustrated in Figure 2. The FORMING voltage was about 1.2 V, the SET and RESET voltages were about +0.6 and −0.75 V, respectively. Figure 3a shows the global statistical distribution of *R*_LRS_ and *R*_HRS_. *R*_LRS_ is distributed in a range of 300 Ω to 1,000 Ω, whereas *R*_HRS_ concentrates around 10 kΩ approximately. To elucidate the influence of LRS on HRS retention properties, the *R*_LRS_ was divided into four groups (marked as ‘G-n’ below) as follows: G-1: 300 Ω to 400 Ω, G-2: 400Ω to 500 Ω, G-3: 500 Ω to 600 Ω, G-4: >600 Ω. The distributions of four groups screened LRS (solid-symbol lines) and corresponding HRS (open-symbol lines) were plotted in Figure 3b. It should be noted that although the LRS were different, the consequent HRS distributions were nearly the same. The reason for this result will be discussed later.

**Figure 4:**
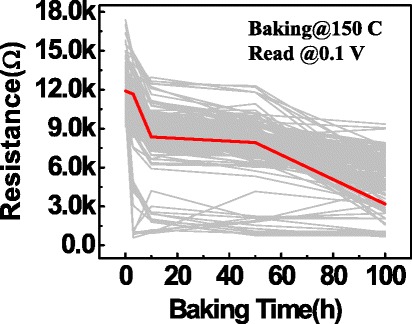
**HRS resistance traces as a function of baking time at 150°C for 1T1R Cu/HfO**_**2**_ **/Pt devices.**

**Figure 5:**
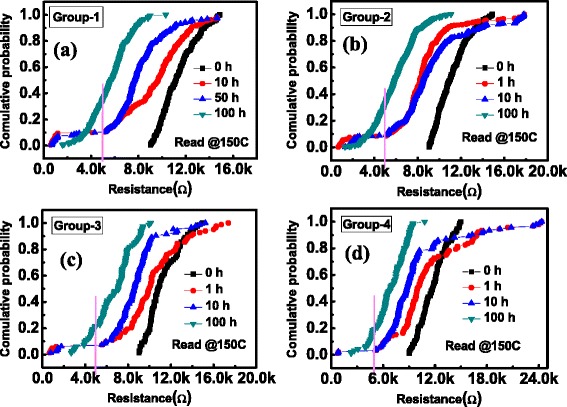
**Cumulative distributions.** Cumulative distributions of *R*_HRS_ for **(a)** G-1, **(b)** G-2, **(c)** G-3, and **(d)** G-4 as the baking time increasing at an annealing temperature *T* = 150°C.

**Figure 6:**
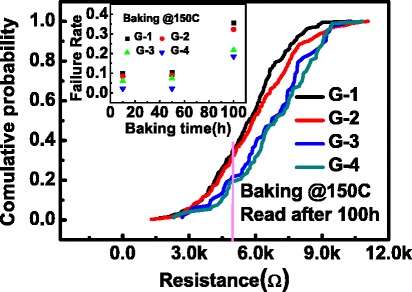
**The probability of** ***R***_**HRS**_ ≤ **5kΩ after baking the same time.** Insert: the failure rate of the *R*_HRS_ decreases as the *R*_LRS_ increases.

The programmed HRS cells were then baked in a vacuum oven at 150°C. The resistance of each cell was periodically checked by a self-made array testing system after cooling down to room temperature. The criterion for judging whether the HRS failed or not was defined by *R*_HRS_ ≤ 5kΩ. Figure 4 shows the dependence of the HRS on the baking time. As the baking time increases, the resistance of the HRS decreases gradually. Figure 5a-d shows the cumulative probabilities of HRS for G-1, G-2, G-3, and G-4, after baking for 0, 10, 50, and 100 h, respectively. Similar evolution trends (shift towards the left) of HRS were found in the four groups as the baking time increased. For the sake of presenting the difference of HRS retention in the four groups clearly, the cumulative probabilities of HRS after a 100-h baking were plotted in one coordinate, as shown in Figure 6. It can be clearly found that the degradation of the HRS programmed from the lowest LRS is worse than that from higher LRS, obeying a relation of G-1 < G-2 < G-3 < G-4. That is to say, the *R*_HRS_ retention is highly dependent on LRS. This result is further supported by summarizing the failure percentage of the HRS at different baking times in the insert of Figure 6.

**Figure 7:**
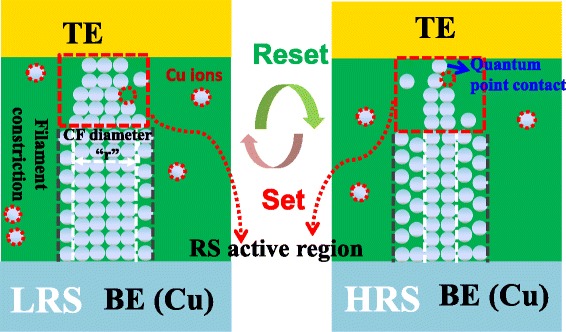
**The schematic of resistive switching mechanism.** According to the quantum point contact (QPC) model, the HRS corresponds to a tiny tunnel gap or neck bridge with atomic size in the filament.

Taking the failure mechanism of retention into account, more attention should be paid on the reconstruction of the conductive filament, which is responsible for the HRS failure behavior (switching to LRS). Based on our previous work [5], the reversible switching between HRS and LRS of Cu/HfO_2_/Pt device is in accordance with the filling and extraction of the copper species from the filament constriction, as sketched in Figure 7. Considering the HRS is in the range from 9 kΩ to 15 kΩ, which is on the level of the quantum conductance, the assumption of the HRS corresponding to a tiny tunnel gap or neck bridge with atomic size in the filament is reasonable according to the quantum point contact (QPC) model [23-25]. Due to the existence of concentration gradient, metal diffusion from the metal-rich filament to the neck point possibly happened, resulting in a gradual decrease of the HRS by the filling or widening of the neck point. In the environment with elevated temperature, the diffusion process was enhanced, leading to the acceleration of the HRS retention failure.

**Figure 8:**
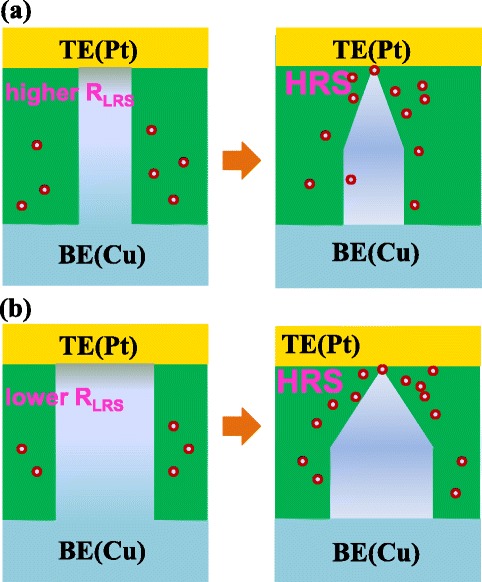
**The explanation for the dependence of HRS retention on different LRS. (a)** The HRS programmed from lower LRS has a smaller residual filament, resulting in slower copper diffusion and better HRS retention. **(b)** On the contrary, the HRS programmed from higher LRS shows the worse retention.

According to the filament theory, the LRS resistance is related closely with the filament size [26,27]. The lower LRS corresponds to the stronger filament. The HRS resistance is mainly determined by the tiny tunnel gap or neck bridge formed during the RESET process. Although the residual filament or the contact resistance may also contribute to HRS resistance, they are much less than the component from the tunnel junction. That is the reason why the HRS of the four groups is at the same level. Figure 8a and b shows the schematic views of HRS programmed from low LRS and high LRS, respectively. Considering both of the cells having a similar HRS, the lengths of tunnel gap or the widths of neck bridge are the same. The main difference for the two cases is the size of the residual filament. Since the metal diffusion from the residual filament to the tunnel gap or neck bridge results in the HRS failure, the case of a smaller residual filament has less probability of gap filling because of the lower copper diffusion, as shown in Figure 8a. In contrast, the HRS with a stronger residual filament gets a higher probability of gap filling, corresponding to worse data retention (Figure 8b). Although the stronger filament can improve the LRS retention, based on the above results, it is not helpful to HRS retention. As a result, we can come to such a conclusion that there exists a balance between the HRS and LRS retention.

## Conclusion

In this letter, we have investigated the influence of LRS on the HRS retention by taking the Cu/HfO_2_/Pt-based CBRAM in a 1-kb 1T1R array as the object. For the retention measurement, the HRS cells which were programmed from different LRS were baked on a vacuum oven under elevated temperature. The results clearly show that the HRS degraded faster as the lowering of the *R*_LRS_. The HRS corresponds to a tiny tunnel gap or neck bridge with atomic size in the filament and would fail to LRS when the gap is filled by the metal diffusion from the residual filament. The lower LRS corresponding to the thicker residual filament, which makes higher probability of gap filling by metal diffusion, results in the worse HRS retention.

## Abbreviations

RRAM: resistive random access memory
HRS: high resistance state
LRS: low resistance state
Cu: copper
Pt: platinum
HfO_2_: hafnium oxide
1 T-1R: one-transistor-one-RRAM
QPC: quantum point contact
*R*_LRS_: resistance of the LRS
ECM: electrochemical metallization mechanism
CBRAM: conductive bridge random access memory
CF: conductive filament
VCM: valence change mechanism
MOS: metal oxide semiconductor
CMP: chemical mechanical polishing
SiO_2_: silicon dioxide
WL: word line
SL: source line
BL: bite line
*R*_HRS_: resistance of the HRS
